# Miscellany

**Published:** 1899-08

**Authors:** 


### MISCELLANY.

The William F. Jenks memorial prize.—The fifth triennial prize of $500, under the deed of trust of Mrs. William F. Jenks, will be awarded to the author of the best essay on The various manifestations of lithemia in infancy and childhood, with the etiology and treatment. This prize is given under the auspices of the College of Physicians of Philadelphia. For details, competitors will address James V. Ingham, M. D., secretary of the trustees.

---

The California Fig Syrup Company has obtained a permanent injunction in the United States Circuit Court, against Clinton E. Worden & Co, of San Francisco, a large non-secret manufacturing house. They are permanently enjoined from using the term syrup of figs or fig syrup, as the name of a laxative medicine which they manufacture, and are required to pay costs and account for sales of the imitations.

---

A good practice for a competent physician is offered in a fine agricultural region in Western New York. The present incumbent has valid reasons for removal and would correspond with an earnest young physician with reference to terms and other details.

Address

Buffalo Medical Journal, 284 Franklin street.

